# Identification of SUMO activating enzyme 1 inhibitors utilizing virtual screening approach

**DOI:** 10.1186/1758-2946-6-S1-P37

**Published:** 2014-03-11

**Authors:** Ashutosh Kumar, Akihiro Ito, Mikako Hirohama, Minoru Yoshida, Kam YJ Zhang

**Affiliations:** 1 Zhang Initiative Research Unit, Institute laboratories, RIKEN, 2-1 Hirosawa, Wako, Saitama 351-0198, Japan; 2 Chemical Genetics Laboratory/Chemical Genomics Research Group, RIKEN, 2-1 Hirosawa, Wako, Saitama 351-0198, Japan

## 

Sumoylation is a post-translational modification affecting diverse cellular processes including DNA replication and repair, chromosome packing and dynamics, genome integrity, nuclear transport, signal transduction and cell proliferation [1]. Sumoylation involves the covalent attachment of a small ubiquitin like modifier (SUMO) protein to ε-amino group of lysine residues in specific target proteins via a sequential action of an activating enzyme E1 (SUMO E1), a conjugating enzyme E2 and a ligase E3. Among the sumoylation proteins, SUMO E1 is responsible for the activation of SUMO in the first step of the sumoylation cascade [2]. SUMO E1 is linked to many human diseases including cancer and thus making it a potential therapeutic target [3]. However, only a few inhibitors were reported up to now that includes three natural products, semi-synthetic protein inhibitors and one AMP mimic [4-6]. Here in this research, the combination of structure based virtual screening and *in vitro* sumoylation assay was used to identify potential small molecule inhibitors of SUMO E1 that could be used in chemical biology and therapeutic studies. Our virtual screening protocol involves the fast docking of a small molecule library to rigid protein followed by redocking of top hits using a method that incorporates both ligand and protein flexibility. Subsequently, the top ranking compounds were prioritized using molecular dynamics simulation based binding free energy calculation. The result of biological assay and subsequent similarity search resulted in the identification of two classes of small molecules that shared biaryl urea scaffold. Both of these chemical classes displayed moderate inhibitory potency against SUMO E1. The most potent compound of each class inhibited the *in vitro* sumoylation with an IC_50_ of 11.1 and 13.4 μM. These compounds inhibit sumoylation by blocking the formation of SUMO-E1 thioester intermediate. Our study presents starting points for the development of novel therapeutic agents against various diseases targeting SUMO E1.
